# From bench to bedside: Development of low-cost, locally fabricated procedural simulation trainers at a hospital-based innovation centre in Pakistan

**DOI:** 10.1016/j.fhj.2026.100571

**Published:** 2026-10-06

**Authors:** Muhammad Abbas Raza, Mohammad Bilawal Khan, Ayaan Ali Qureshi, Ghalib Ali, Ghansham Bhagwan Das, Abdul Ameen, Selina Hasan, Faisal Wasim Ismail

**Affiliations:** a Department of Medicine, Aga Khan University Hospital, Stadium Road, Karachi, 74800, Pakistan; b Medical College, Aga Khan University, Stadium Road, Karachi, 74800, Pakistan; c Centre for Innovation in Medical Education (CIME), Aga Khan University Hospital, Stadium Road, Karachi, 74800, Pakistan

**Keywords:** Simulation-based medical education, Low-cost simulation, LMIC, Procedural training, Medical device fabrication, Pakistan

## Abstract

**Background:**

Simulation-based medical education is central to procedural skills training, but commercial simulators remain financially inaccessible to many institutions in low- and middle-income countries (LMICs). The Centre for Innovation in Medical Education (CIME) at Aga Khan University, Pakistan, developed four low-cost, locally fabricated procedural simulation trainers to address this gap.

**Methods:**

This technical innovation report describes the design, material selection, fabrication methodology, and cost analysis of a Fiberoptic Intubation Box, a Heel Prick Simulator, a Haemorrhoid Ligation Simulator, and a Caesarean-Section Laparotomy Model, developed using locally available or repurposed materials through faculty-guided iterative prototyping.

**Results:**

Each device achieved greater than 95% cost reduction versus commercial equivalents, including a reduction from USD $12,000 to USD $4 for the laparotomy model, while retaining features supporting authentic procedural practice.

**Conclusions:**

These devices demonstrate that LMICs can develop high-utility procedural simulators at a fraction of commercial cost, with open-access blueprints enabling global replication.

## Highlights

● Four low-cost simulators were built at a Pakistan hospital, cutting cost by >95%.
● A five-phase, faculty-guided process was used to design all four trainers.
● Heel Prick Simulator enables authentic puncture with controlled blood flow.
● Haemorrhoid trainer reuses discarded IV-arm silicone at near-zero material cost.
● Open-access fabrication blueprints enable replication in other LMICs.

## Introduction and background

Simulation-based medical education (SBME) is a cornerstone of procedural skills training, improving procedural competence and patient safety before trainees encounter real patients.^1^, ^2^ Commercial high-fidelity simulators for procedures such as fiberoptic airway management, neonatal blood sampling, anorectal interventions, and obstetric surgery are expensive, typically ranging from GBP £367/USD $500 to over GBP £8,800/USD $12,000 per unit.^3^ A landmark BEME review identified high-fidelity simulation as effective when it includes deliberate practice, feedback, and curriculum integration,^4^ and subsequent meta-analyses confirm this across clinical tasks.^5^, ^6^ Despite this evidence, global adoption of SBME remains inequitable, with resources concentrated in high-income countries,^7^, ^8^ an inequity particularly acute for procedural simulation, which requires physical task trainers or manikins rather than software.

Pakistan, a lower-middle-income country (LMIC) per the World Bank,^9^ faces a well-documented procedural training gap attributable to the cost and limited availability of simulation equipment.^10^ For LMICs like Pakistan, these costs are an insurmountable barrier. Healthcare professionals outside major urban centres frequently perform these procedures without simulation-based preparation, risking patient safety,^11^ despite high demand for simulation in areas such as airway emergencies, neonatal metabolic screening, anorectal disease, and obstetric emergencies.^12^

A growing literature documents low-cost simulation devices fabricated from household materials and repurposed medical waste for procedures including venipuncture, catheterisation, suturing, and obstetric simulation.^13^, ^14^, ^15^ However, published reports commonly lack complete fabrication details, and few originate from South Asia. The Centre for Innovation in Medical Education (CIME) at Aga Khan University, Karachi, was established to address this gap^16^; its Technical Team, comprising biomedical engineers and clinical faculty, developed four low-cost trainers targeting fiberoptic intubation, neonatal heel prick sampling, haemorrhoid ligation, and Caesarean section laparotomy. Our objectives were: (1) to describe a replicable technical innovation process for other LMIC institutions; (2) to provide open-access fabrication blueprints; and (3) to contribute a South Asian institutional experience to this literature.^17^ All four devices achieved greater than 95% cost reduction versus commercial equivalents while maintaining sufficient anatomical fidelity for meaningful procedural training. Although developed in a resource-limited setting, these fabrication approaches also offer practical value for high-income country programmes seeking to expand simulation access, reduce training costs, and adopt more sustainable, reusable teaching materials amid growing pressures on medical education budgets.

## Methods

### Development framework

All four simulators were developed within the CIME Technical Laboratory at Aga Khan University between 2023 and 2025, using a shared five-phase framework^8^: (1) needs assessment and commercial benchmarking; (2) design requirements; (3) material evaluation and iterative prototyping; (4) structured faculty/trainee feedback; and (5) finalisation of fabrication protocol and cost analysis. The BronchBox additionally employed a modified V-model^18^ for systematic design and verification. Three-dimensional modelling in Autodesk Fusion 360 supported digital validation before fabrication. A micro-costing approach estimated procurement and per-procedure consumable costs, excluding existing institutional assets (eg, fiberoptic scope, repurposed manikins). Cost reduction was calculated as (Commercial Cost − In-House Cost)/Commercial Cost × 100, with costs converted from PKR to USD and GBP at USD 1 = PKR 280 and GBP 1 = PKR 378.

### Material selection principles

Materials were selected according to four criteria: anatomical realism, cost-effectiveness, local market availability, and reusability. Repurposed or recycled materials were prioritised where possible. Prototypes were iteratively modified following review sessions with faculty and trainees, and all materials were procured from the local Karachi market to ensure reproducibility without reliance on international supply chains.

### Ethical considerations

This was a technical developmental project; no human participants, patient data, biological specimens, or personal information were involved, and no identifiable person appears in any image. The study was reviewed by the Aga Khan University Ethics Review Committee and granted exemption from ethical approval (ERC No. 2026-13130-42182).

## Device 1: Fiberoptic Intubation Box (BronchBox)

### Clinical rationale and needs assessment

Fiberoptic intubation is essential for anaesthesia providers managing difficult airways, requiring hand-eye coordination and scope navigation that cannot be acquired through didactic instruction alone.^19^, ^20^ Commercial fiberoptic teaching tools are widely used in high-income countries but are prohibitively expensive for most LMICs.^21^ The BronchBox was developed at CIME between September and December 2023, inspired by the Oxford Fiberoptic Teaching Box concept^22^ and adapted to the Pakistani material and budgetary context. The device aims to replicate the essential spatial and dexterous challenges of scope navigation at a fraction of commercial cost.^23^

### Design requirements

The device was required to replicate the spatial challenges of scope navigation in a standardised, modular environment allowing progressively increasing difficulty; to be safe for delicate fiberoptic equipment, with all scope-contact surfaces smooth and burr-free; and to be portable and fabricable from locally available materials.

### Material selection

Acrylic (polymethyl methacrylate, PMMA) was selected as the primary structural material after evaluating polycarbonate and PVC, for its tensile strength (70 MPa), elastic modulus (2.7 GPa), impact resistance five to six times greater than glass, optical clarity, and local low-cost availability. Plates were specified at 25.4 × 25.4 cm and 0.5 cm thickness. Laser cutting was used instead of mechanical drilling, which produced rough, burred edges risking scope damage; laser cutting produced clean, precise edges via a local fabrication service. Table 1 presents the material breakdown.

**Table 1:** BronchBox component specifications.

Component	Material used	Function/purpose
Structural box frame	Acrylic (PMMA) sheets, 0.5 cm thickness	Provides the vertical housing (45 cm in height, 25 × 25 cm base) for the plate assembly
Navigation plates (×6)	Acrylic (PMMA), 25 × 25 cm, 0.5 cm thickness	Six interchangeable plates with laser-cut circular and plus-shaped holes of progressive difficulty
Hole cutting	Laser-cut (local fabrication service)	Produces smooth, burr-free holes to prevent damage to the fiberoptic scope tip
Mounting supports	Internal acrylic brackets	Secures plates at fixed 7.5 cm intervals within the frame
Side access panel	Removable acrylic panel with fixings	Allows plate exchange and reconfiguration for modular difficulty levels
Digital design file	Autodesk Fusion 360 CAD model	Used for digital design validation of plate hole configurations prior to fabrication

### Device design and specifications

The BronchBox comprises a vertical acrylic box (45 cm in height, 25 × 25 cm in base) housing six horizontally mounted plates spaced 7.5 cm apart, each with a unique configuration of laser-cut circular and plus-shaped holes of progressive difficulty, from simpler early configurations to finer scope manipulation later in the sequence. One side panel is removable for plate reconfiguration, making the system modular. The design was validated digitally in Autodesk Fusion 360 before fabrication. The final product is shown in Fig. 1.

**Fig. 1:**
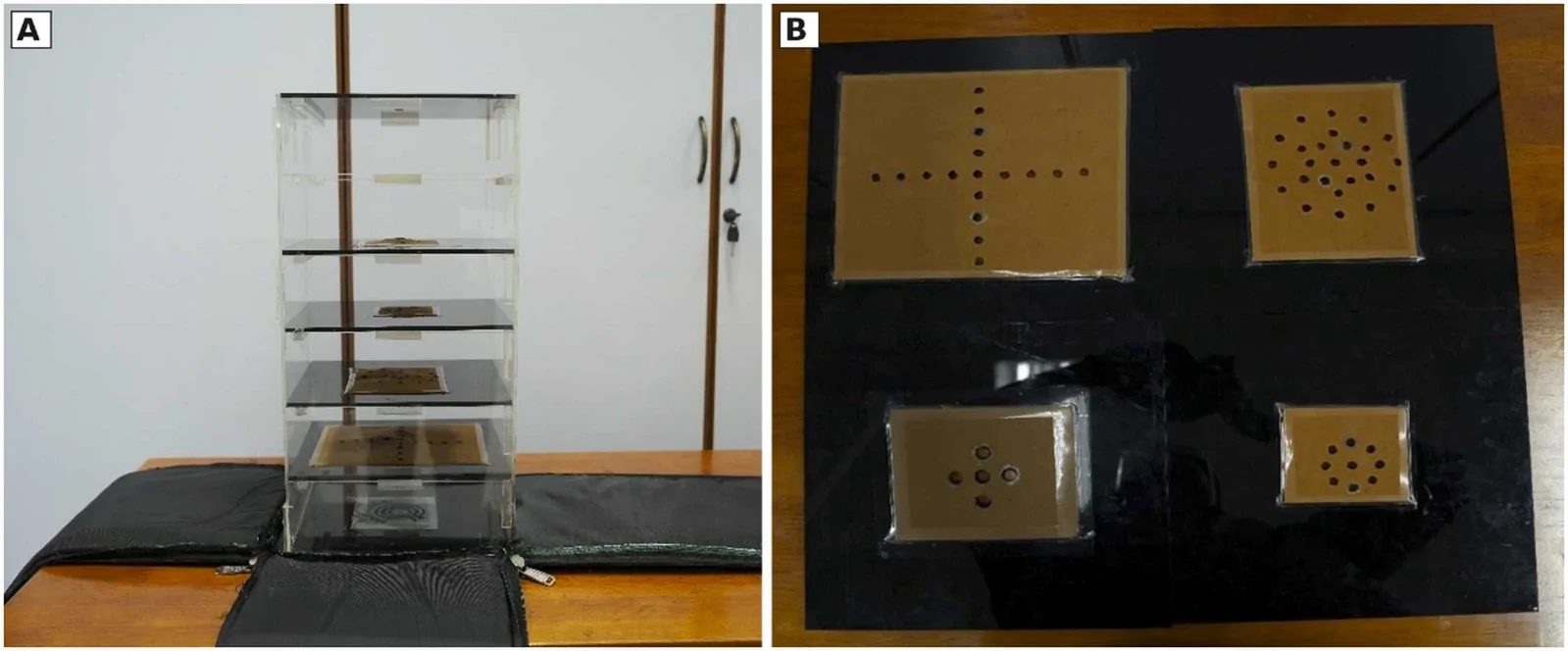
Fiberoptic Intubation Box (BronchBox): assembled device (A) and close-up of laser-cut acrylic plate hole configurations (B).

### Fabrication protocol

**Step 1:** Cut six acrylic plates to 25 × 25 cm dimensions at 0.5 cm thickness using a table saw or laser cutter.

**Step 2:** Design hole configurations for each plate in Autodesk Fusion 360 or equivalent CAD software, incorporating circular and plus-shaped patterns of increasing difficulty across plates 1–5 (Fig. 2).

**Fig. 2:**
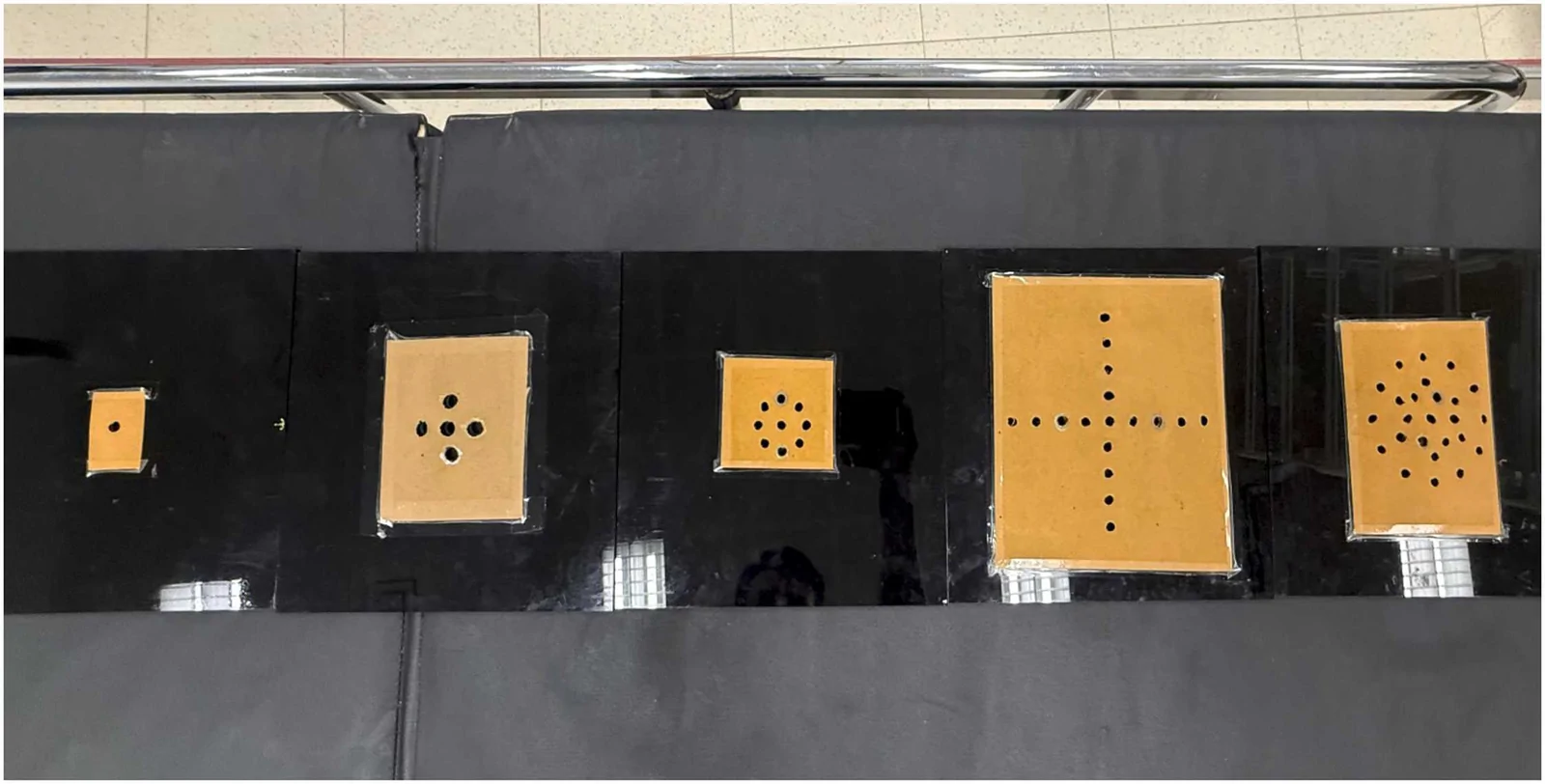
BronchBox plate progression: plates 1–5 demonstrating increasing hole complexity from novice to expert level.

**Step 3:** Laser-cut all holes according to the finalised design; verify edge smoothness before assembly.

**Step 4:** Construct the acrylic box frame (45 cm in height, 25 × 25 cm in base) with internal mounting supports at 7.5 cm intervals.

**Step 5:** Insert plates into the frame at the designated 7.5 cm intervals, ensuring each plate is level and firmly seated.

**Step 6:** Secure one side panel with removable fixings to allow plate exchange and reconfiguration.

**Step 7:** Conduct a final inspection to confirm all internal surfaces are smooth and free of protrusions that could damage a fiberoptic scope.

### Additional refinements

Iterative faculty and trainee feedback refined hole sizing (a two-tier sizing protocol was added for novice users) and plate spacing, which was increased from 5 to 7.5 cm to better mimic the depth of anatomical challenge encountered clinically. The refined plates are shown in Fig. 2.

### Cost analysis

Compared with leading commercial simulation manufacturers, the BronchBox achieves >95% cost reduction (Table 2).^24^

**Table 2:** BronchBox (Fiberoptic Intubation Box) cost analysis.

Cost item	Unit cost (PKR/GBP/USD)
**Total In-House Cost**	PKR 30,000/GBP £79/USD $107
**Commercial Equivalent**	PKR 893,200/GBP £2,363/$USD 3,190^24^
**Estimated Saving**	>95%

PKR, Pakistani Rupee; GBP, Great British Pound; USD, United States Dollar. Cost reduction calculated as (Commercial Cost − In-House Cost)/Commercial Cost) × 100. This commercial equivalent (Laerdal Airway Management Trainer) is not the same design but represents a commercially available airway management trainer of comparable functional category.

## Device 2: Heel Prick Simulator

### Clinical rationale and needs assessment

Neonatal heel prick (capillary blood sampling) is a universal newborn screening procedure detecting disorders including phenylketonuria, congenital hypothyroidism, and haemoglobinopathies,^25^ requiring precise lancet technique to achieve correct puncture depth (2 mm) while minimising discomfort and sample haemolysis. Healthcare professionals often receive no hands-on training beforehand, and no known commercial device enables authentic lancet puncture with complete filter paper collection. The Heel Prick Simulator (Fig. 3) was developed to replicate this complete workflow.

**Fig. 3:**
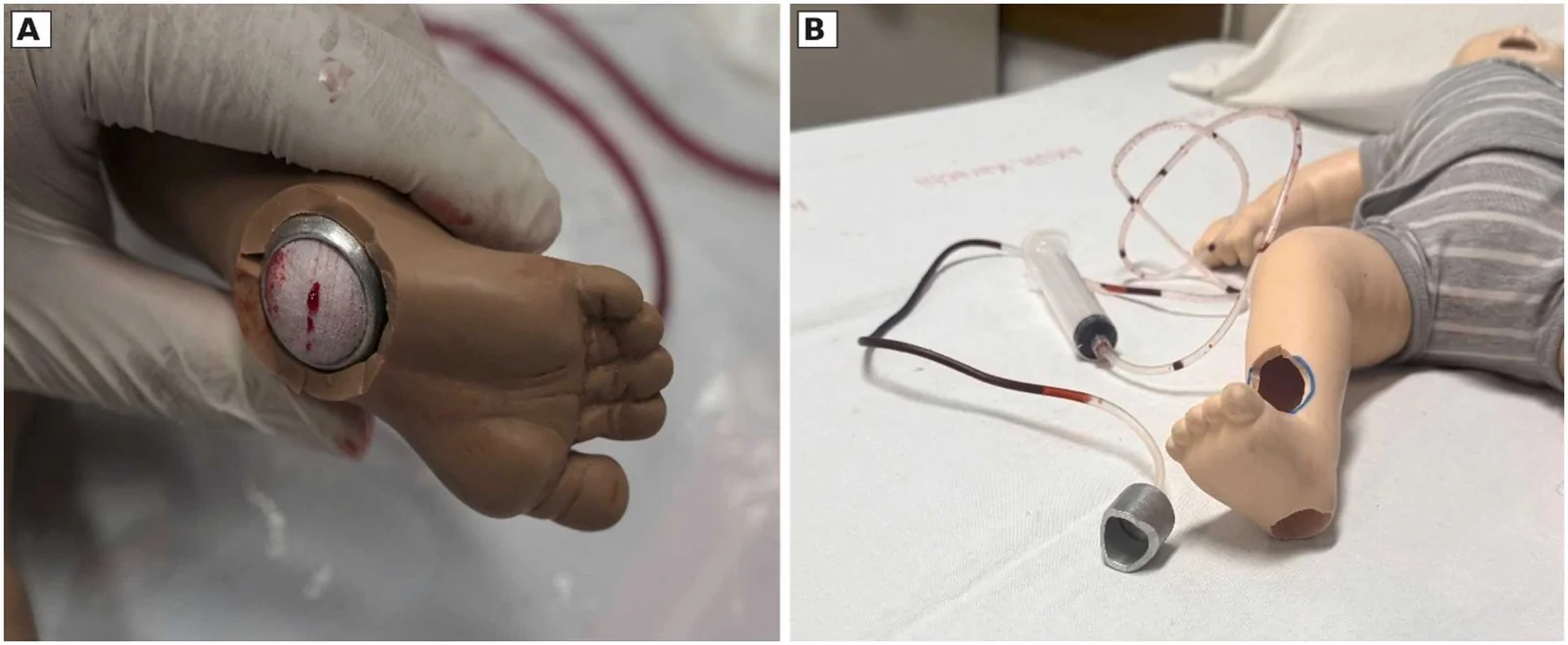
Heel Prick Simulator: assembled device installed in baby manikin (A); aluminium heel shaper with drip-set connection (B).

### Material evaluation and iterative prototyping

Six candidate skin-layer materials were systematically evaluated for their ability to mimic a 2 mm lancet puncture with realistic tactile feedback and controlled blood flow (Table 3).

**Table 3:** Prototyping iterations for the Heel Prick Simulator skin layer.

Prototype material	Outcome/reason for rejection
Surgical gloves	Insufficient tactile realism; did not replicate puncture feel or neonatal skin tension
3D-printed skin	Limited success in mimicking puncture sensation; inadequate blood collection capability
PVC bags	Did not replicate required 2 mm puncture depth accurately
Unstructured cotton padding	Lacked structural integrity for repeatable training use
Silicone tubes	Insufficient puncture realism; poor tactile feedback
Diaper outer layer + cotton + aluminium shaper (FINAL)	Optimal combination: realistic skin tension, correct puncture depth, controlled blood flow

### Final device design and components

The finalised simulator comprises four integrated components working together to replicate the complete procedural workflow (Table 4).

**Table 4:** Heel Prick Simulator component specifications.

Component	Material	Function
Skin layer	External waterproof layer of a commercial diaper	Simulates neonatal skin; allows authentic 2 mm lancet puncture while preventing fluid leakage
Subcutaneous padding	Cotton swab/cotton ball	Provides tissue-like compression beneath the skin layer, replicating subcutaneous tissue
Heel shaper	Custom-fabricated aluminium	Provides anatomical heel geometry; lid connects securely to drip-set tube to prevent leakage
Blood delivery system	IV drip-set tube + 60 mL syringe	Operator controls simulated blood flow via syringe plunger, simulating capillary emergence
Simulated blood	Glycerine (50 mL) + food-grade blood concentrate (10 mL)	5:1 ratio replicates viscosity, colour, and flow of neonatal capillary blood
Training platform	Baby manikin	Provides realistic anatomical context for heel positioning and procedure practice

### Simulated blood formulation

A simulated blood mixture combining 50 mL glycerine with 10 mL food-grade blood concentrate (5:1 ratio), replicates the viscosity, colour, and flow of neonatal capillary blood and is visually indistinguishable from blood on filter paper (Fig. 4).

**Fig. 4:**
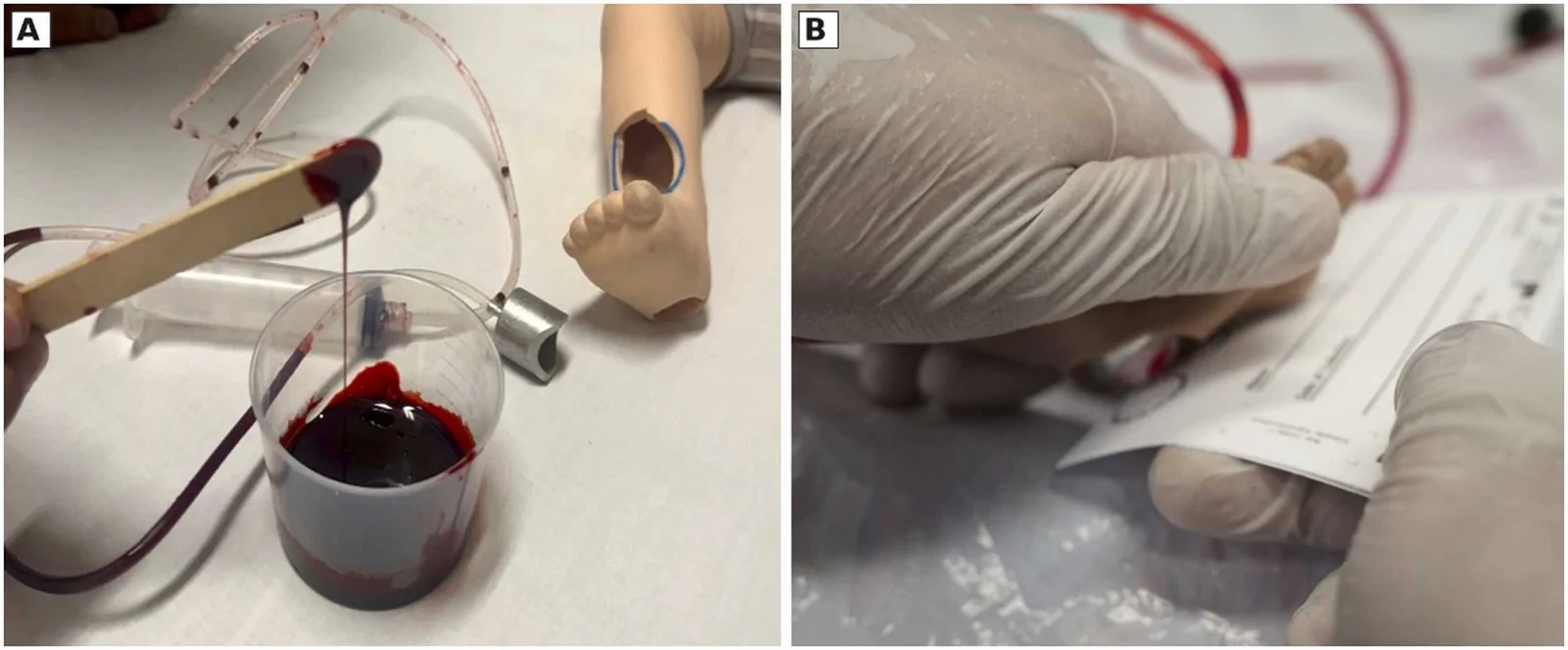
Simulated blood formulation preparation: glycerine and blood concentrate mixed (A); blood spot collection on filter paper card (B).

### Fabrication protocol

**Step 1:** Measure and combine 50 mL of pure glycerine with 10 mL of food-grade blood concentrate in a clean container.

**Step 2:** Take a cotton swab/ball and wrap it with the external waterproof layer excised from a commercial diaper, securing tightly.

**Step 3:** Fix the wrapped cotton assembly into the custom-fabricated aluminium heel shaper, conforming to the anatomical heel geometry.

**Step 4:** Connect one end of an IV drip-set tube to the back lid of the aluminium shaper (Fig. 3); alternatively, thread the tube internally through the cotton and secure with thread to prevent leakage.

**Step 5:** Load a 60 mL syringe with the prepared simulated blood solution and connect to the free end of the drip-set tube.

**Step 6:** Insert the complete assembly (heel shaper with skin layer) into the heel of the baby manikin. The simulator is ready for use.

### Usage and key innovations

The trainee uses a 2 mm lancet to prick the skin surface at the anatomically correct heel site; the facilitator controls blood flow via the syringe plunger, and the trainee collects droplets onto filter paper (Fig. 4), replicating the complete neonatal screening workflow. The syringe mechanism allows flow rate to be varied to simulate different clinical scenarios. To the authors' knowledge, no published Heel Prick Simulator combines authentic lancet puncture, operator-controlled blood flow, and filter paper collection in a single platform.

### Cost analysis

See Table 5.

**Table 5:** Heel Prick Simulator cost analysis.

Cost Item	Unit Cost (PKR/GBP/USD)
**Total In-House Cost**	PKR 6,950/GBP £18/USD $25
**Commercial Equivalent**	PKR 117,936/USD $421.20/GBP £312^26^
**Estimated Saving**	>95%

PKR, Pakistani Rupee; GBP, Great British Pound; USD, United States Dollar. Commercial equivalent: Newborn Blood Spot Test Simulator (Sim & Skills, UK).

## Device 3: Haemorrhoid Ligation Simulator

### Clinical rationale and needs assessment

Rubber band ligation of internal haemorrhoids is one of the most common office-based procedures in general surgery and gastroenterology, used to treat symptomatic grade I to III internal haemorrhoids.^27^ It requires hands-on familiarity with anorectal anatomy, anoscope insertion, and tissue engagement best acquired with a physical trainer. Commercial simulators exist but are costly and largely inaccessible to LMICs.^28^ This project added a further innovation: the deliberate use of repurposed medical waste as fabrication material, addressing training access and sustainability together.

### Material innovation: repurposed medical waste

The defining innovation is the use of silicone skin excised from discarded IV arm trainers, retired once their cannulation surface is too perforated for further use, but whose underlying silicone retains structural integrity. This otherwise-discarded material provides a realistic analogue for rectal mucosal tissue that withstands repeated instrument insertion. The costs associated with this component, including procurement of the silicone material and technician time for excision, cleaning, and preparation, are included in the cost analysis below (Section 5.7).

### Device design and components

See Table 6.

**Table 6:** Haemorrhoid Ligation Simulator component specifications.

Component	Material used	Anatomical structure simulated
Rectal mucosal surface	Silicone skin from discarded IV arm trainers	Rectal mucosa with realistic texture and insertion resistance
Intestinal lumen	Plastic pipes	Rectal canal through which the anoscope and ligator are passed
Haemorrhoidal tissue	Medical-grade silicone	Internal haemorrhoids at anatomically correct positions
Structural frame	Reinforced frame construction	Stability for repeated instrument insertion without displacement
Procedure instrument	Standard rubber band ligator	Realistic tool–tissue interaction for ligation training

### Fabrication protocol

**Step 1:** Procure discarded IV arm trainers from the simulation laboratory waste stream; excise the silicone skin sheets and clean thoroughly.

**Step 2:** Shape the silicone into a cylindrical rectal canal of anatomically appropriate diameter, securing overlapping edges.

**Step 3:** Cut plastic pipes to the required length and diameter to form the intestinal lumen; insert into the silicone canal.

**Step 4:** Fabricate and attach three medical-grade silicone haemorrhoids at the 3, 7, and 11 o'clock positions within the lumen (Fig. 5).

**Fig. 5:**
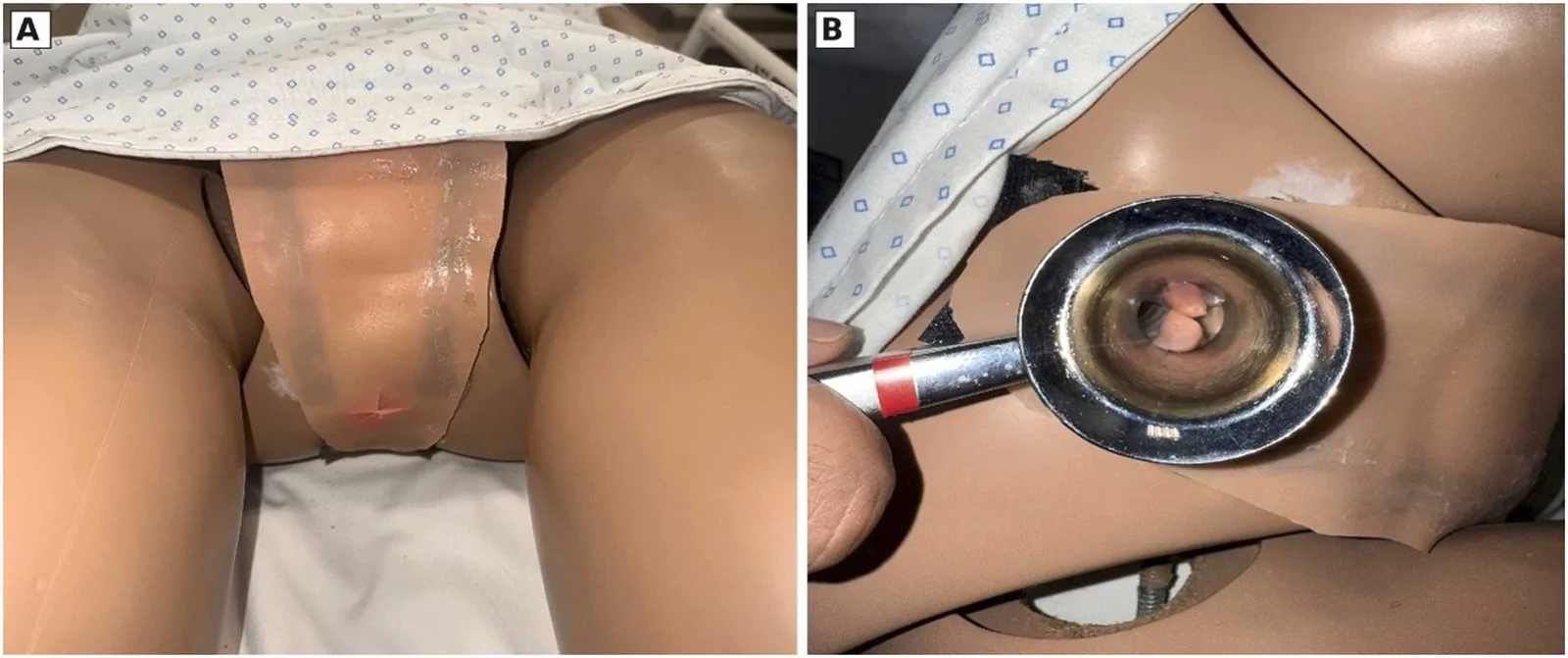
Haemorrhoid Ligation Simulator: external view (A); cross-section showing lumen and medical-grade silicone haemorrhoids at anatomically correct positions (B).

**Step 5:** Mount the complete assembly in a reinforced structural frame that provides stable resistance during anoscope insertion and ligator deployment.

**Step 6:** Verify that the standard rubber band ligator can be advanced, positioned, and fired within the lumen without structural displacement.

### Additional refinements

Feedback from colorectal surgery faculty led to substitution of lower Shore-hardness medical-grade silicone for improved tissue realism, and adjustment of lumen diameter to better match clinical anoscopy dimensions (Fig. 6).

**Fig. 6:**
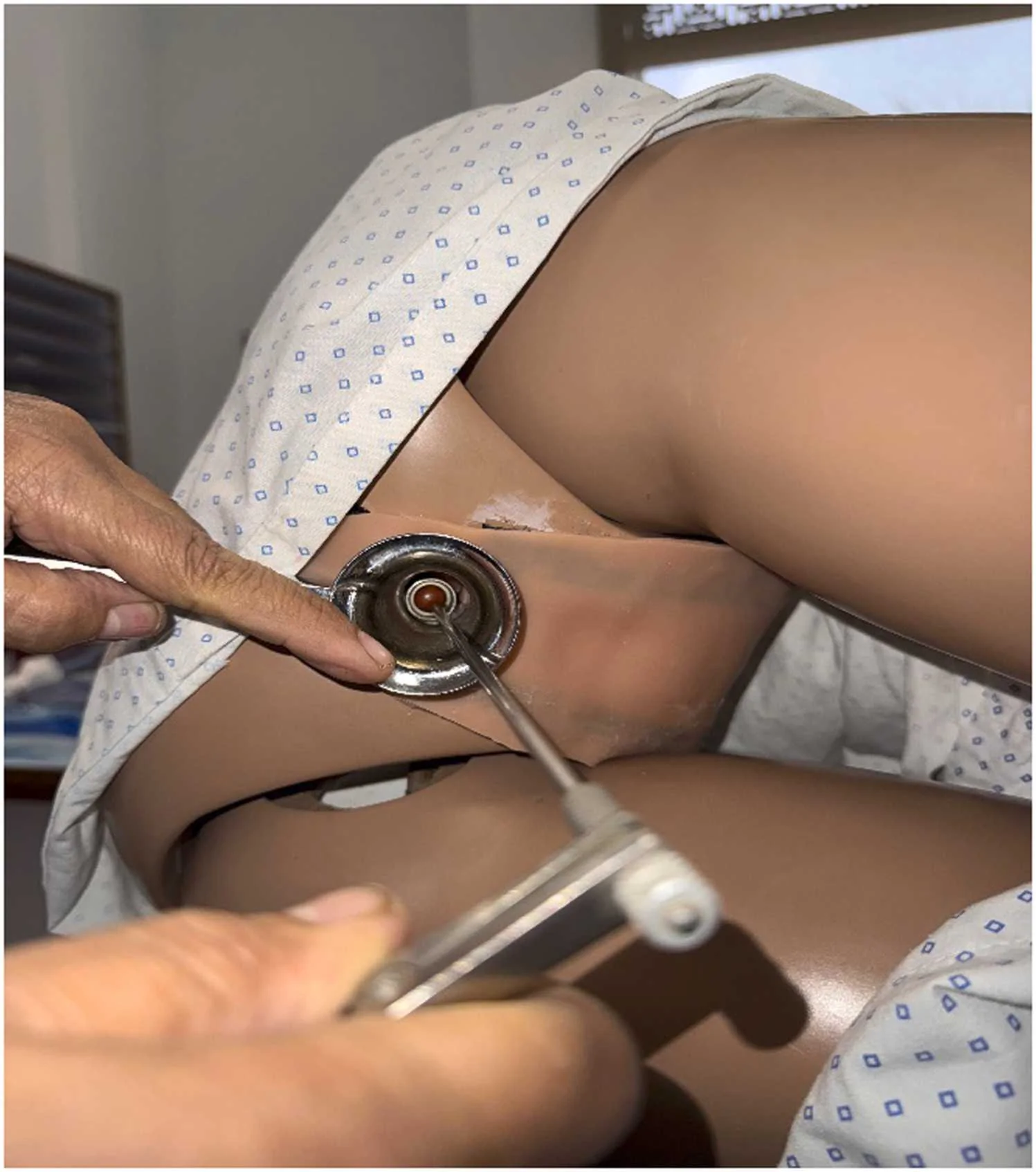
Haemorrhoid Ligation Simulator in use: rubber band ligator positioning over haemorrhoid.

### Sustainability and design innovation

Sourcing the material entirely from laboratory waste reduces both cost and disposal burden, showing that circular-economy principles apply to simulation fabrication; to our knowledge, no commercial Haemorrhoid Ligation Simulator uses a comparable repurposed-waste design.

### Cost analysis

See Table 7.

**Table 7:** Haemorrhoid Ligation Simulator cost analysis.

Cost item	Unit cost (PKR/GBP/USD)
**Total In-House Cost**	PKR 1,400–2,800 (USD 5–10/GBP 3.70–7.41)^a^
**Commercial Equivalent**	PKR 770,000**/**GBP £2,020/USD $2,750^29^^a^
**Estimated Saving**	>95%

a This simulator was fabricated entirely from repurposed materials (silicone from retired IV arm trainers); if sourced this way, the raw material cost would be near-zero. The nearest commercial alternative (Chamberlain Group Haemorrhoidal Banding Trainer, GBP £2,020) differs substantially in design concept and materials. PKR, Pakistani Rupee; GBP, Great British Pound; USD, United States Dollar.

## Device 4: Caesarean Section Laparotomy Model

### Clinical rationale and needs assessment

Caesarean section is amongst the most commonly performed surgical procedure globally, and perimortem Caesarean delivery is among the most time-critical obstetric interventions,^30^ requiring familiarity with incision technique, layer-by-layer dissection, uterine entry, and haemorrhage management. Simulation training is an important adjunct for junior trainees,^31^ but commercial models can cost approximately GBP 8,800/USD $12,000/PKR 3,331,000.^32^ The CIME laparotomy model (Fig. 7) was developed to bridge this gap, supporting the emergency obstetric workshop at Aga Khan University.

**Fig. 7:**
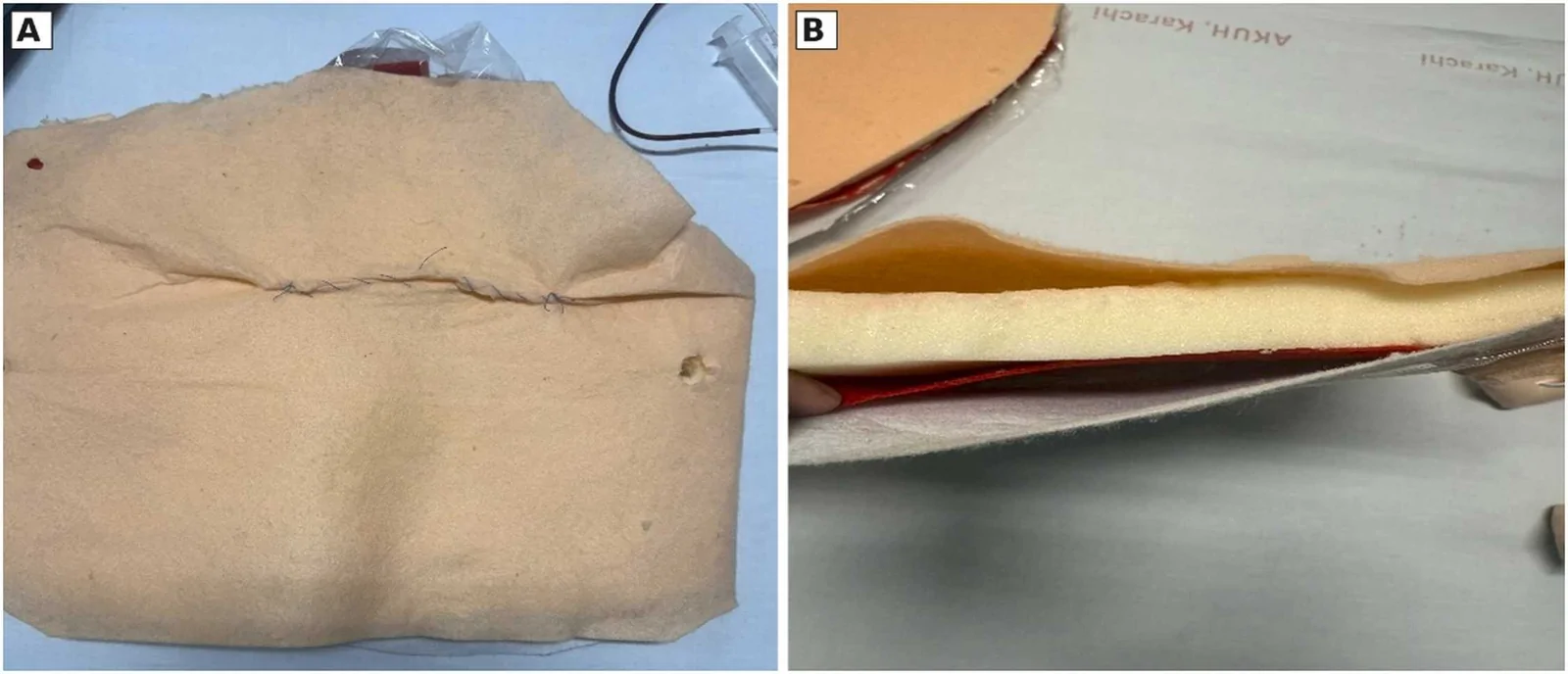
C-Section Laparotomy Model: assembled model showing skin surface (A); cross-section demonstrating anatomical layers (B).

### Anatomical layer specifications

The device replicates six anatomical layers of the lower abdominal wall and uterus (Table 8, Fig. 7).

**Table 8:** C-Section Laparotomy Model: anatomical layer specifications.

Layer	Anatomical structure	Material used	Colour
1	Skin	Felt sheet	Flesh/skin tone
2	Subcutaneous fat	Foam sheet	White
3	Rectus abdominis muscle	Felt sheet	Red
4	Rectus sheath/fascia	Transparent vinyl sheet	White/transparent
5	Peritoneum	Transparent vinyl sheet	Transparent
6	Uterine wall (myometrium)	Felt sheet	Red

### Blood flow simulation system

Airtight IV drip-set tubing is embedded between layers during assembly and pre-filled with the simulated blood solution described for Device 2. Incision through the tubing releases pressurised simulated blood, replicating intraoperative haemorrhage and enabling training in haemorrhage recognition, haemostatic manoeuvres, and blood management not otherwise rehearsable in dry-run models.

### Fabrication protocol

**Step 1:** Cut each material layer (felt, foam, vinyl) to the appropriate dimensions replicating the lower abdominal wall surface area.

**Step 2:** Prepare the simulated blood solution (50 mL glycerine + 10 mL blood concentrate per reservoir).

**Step 3:** Thread IV drip-set tubing in a branching vascular pattern between layers 3 and 4 (muscle and fascia); ensure tubing is airtight and connected to a filled reservoir (Fig. 8).

**Fig. 8:**
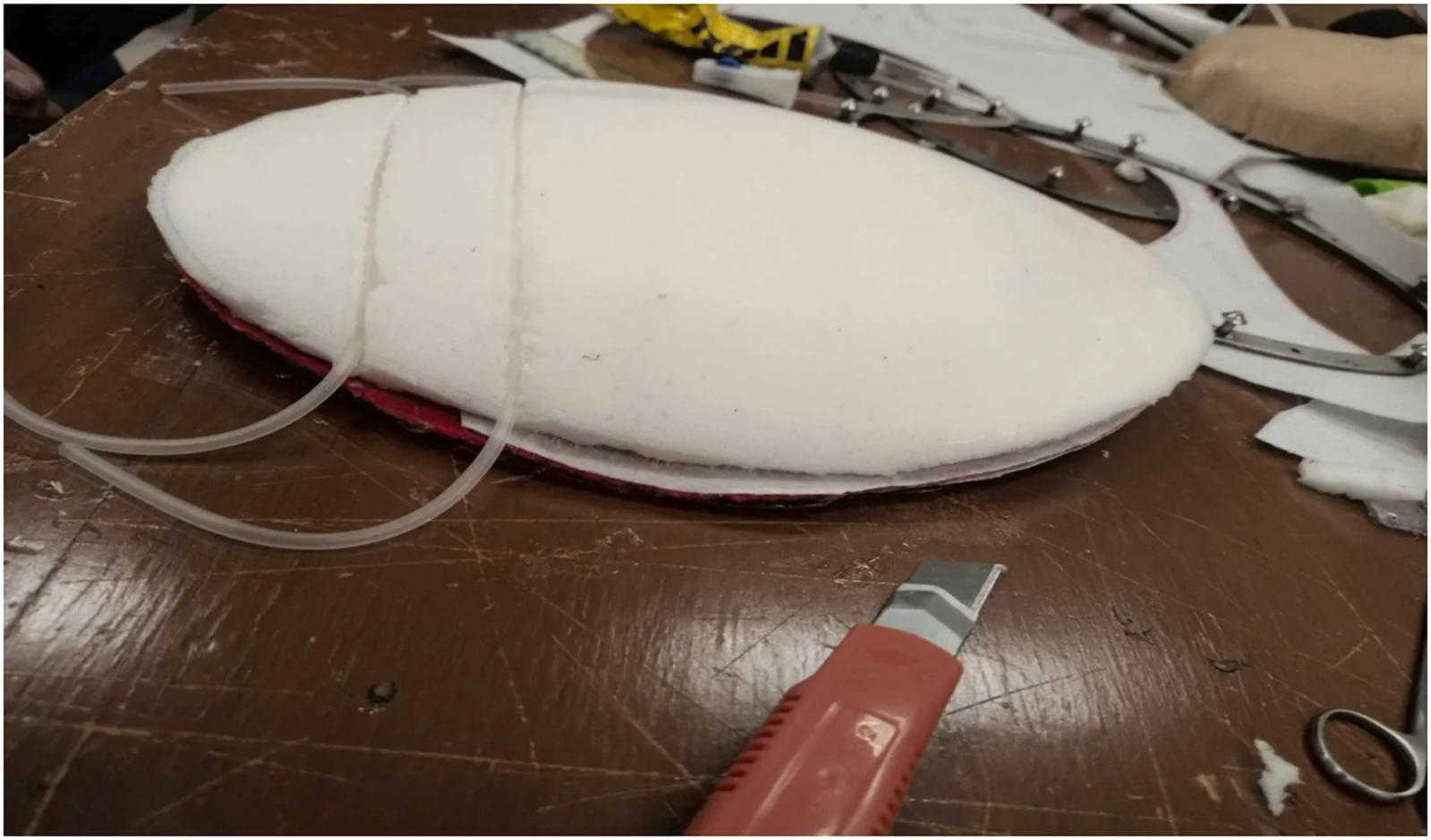
IV drip-set tubing embedded between layers during assembly.

**Step 4:** Assemble the six layers sequentially, adhering each layer to the next using appropriate fabric adhesive.

**Step 5:** Confirm that the completed assembly allows a scalpel to pass through each layer sequentially with appropriate resistance and check that each layer can be identified tactilely and visually during dissection.

**Step 6:** Test the blood flow system by making a controlled incision to the tubing layer; verify blood flow onset and flow rate.

**Step 7:** Rebuild the incised model for reuse: replace incised felt layers, re-thread tubing, and re-adhere layers as needed.

### Additional refinements

Faculty feedback led to substitution of a lower-density foam for the subcutaneous fat layer, thickening of the felt muscle layer for realistic dissection resistance, and refinement of blood-flow tubing routing for a more realistic haemorrhage pattern.

### Cost analysis

See Table 9.

**Table 9:** C-Section Laparotomy Model cost analysis.

Cost Item	Unit Cost (PKR/GBP/USD)
**Total In-House Cost**	PKR 1,153/GBP £3/USD $4
**Commercial Equivalent**	Approximately PKR 3,360,000/GBP £8,889/USD $12,000^32^
**Estimated Saving**	>95%

No single commercial equivalent was identified for direct comparison. The commercial reference (Anatomy Warehouse Wearable C-Section Simulator, ~GBP £8,800) differs in design concept. Costs were calculated on a per-model basis using locally procured materials from Karachi, Pakistan. PKR, Pakistani Rupee; GBP, Great British Pound; USD, United States Dollar.

## Cross-project summary and comparative overview

Table 10 provides a consolidated comparison of the four simulation devices across key dimensions: procedure trained, primary materials, key technical innovation, and cost reduction achieved (Fig. 9).

**Table 10:** Cross-project comparative overview of four CIME simulation devices.

Device	Procedure trained	Key material innovation	Cost saving vs. commercial
Fiberoptic Intubation Box (BronchBox)	Fiberoptic airway scope navigation	Laser-cut acrylic; modular six-plate progressive difficulty; 3D modelled in Autodesk Fusion 360	>95% vs. Laerdal Airway Management Trainer (GBP £2,363/USD $3,190/PKR 893,200).^24^
Heel Prick Simulator	Neonatal capillary blood sampling (heel prick)	Diaper outer layer (skin); aluminium heel shaper; IV drip-set with operator-controlled glycerine-based blood; filter paper collection	>95% vs. GBP £312/USD $421.20/PKR 117,936^26^
Haemorrhoid Ligation Simulator	Rubber band ligation of internal haemorrhoids	Repurposed silicone from discarded IV arm trainers (medical waste); medical-grade silicone haemorrhoids	>95% vs. Chamberlain Group Haemorrhoidal Banding Trainer (GBP £2,037/USD $2,750/PKR 770,000)^29^
C-Section Laparotomy Model	Caesarean section incision, dissection, haemorrhage management	Six-layer felt/foam/vinyl abdominal wall; airtight IV drip-set blood flow simulation on incision	>95% vs. GBP £8,889/USD $12,000/PKR 3,360,000^32^

**Fig. 9:**
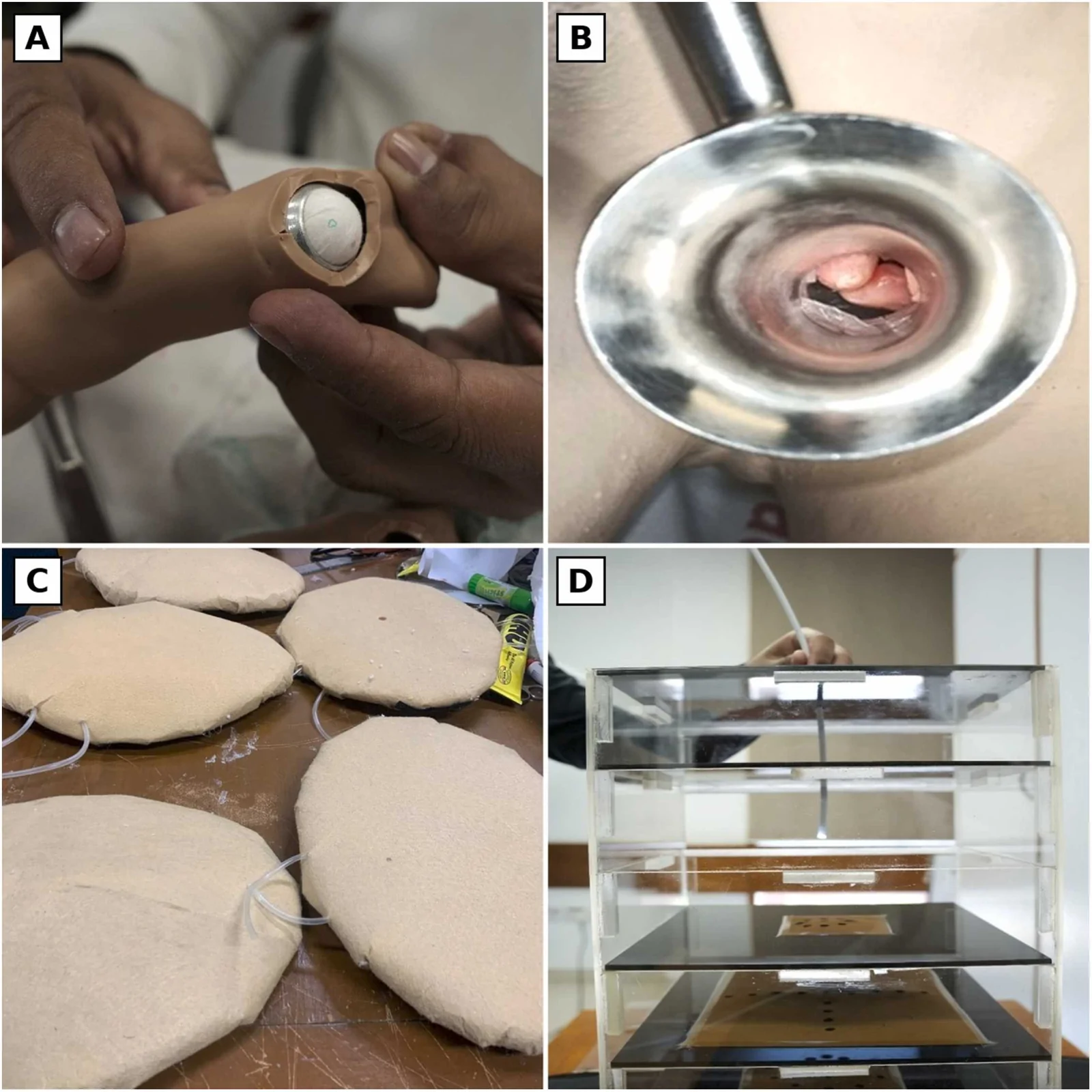
All four CIME simulation devices: BronchBox (A), Heel Prick Simulator (B), Haemorrhoid Ligation Simulator (C), C-Section Laparotomy Model (D).

## Discussion

### Principal findings, scalability and replication

We developed four low-cost procedural simulation trainers, reducing production costs by more than 95% versus commercial equivalents. The devices address high-priority training gaps representing a significant share of the procedural competency demands on LMIC healthcare systems, consistent with the Global Consensus Statement on Simulation-Based Practice in Healthcare, which emphasises contextual adaptation and accessible solutions.^33^

Iterative, faculty-guided prototyping was central to achieving anatomical fidelity: no device reached its final form in fewer than three cycles, and key improvements, such as the BronchBox laser-cutting decision and the Heel Prick Simulator's aluminium shaper, emerged from structured clinical feedback rather than engineering planning alone. The Heel Prick Simulator is a particularly novel contribution: to our knowledge, no published or commercial device combines authentic 2 mm lancet puncture, operator-controlled blood flow, and filter paper collection in one platform. The Haemorrhoid Ligation Simulator demonstrates that repurposed silicone from retired IV arm trainers can provide clinically acceptable material at near-zero raw material cost. Total cost includes technician time for preparation and processing, with silicone procurement required when repurposed material is unavailable. Departments with similar retired equipment could replicate this approach, while others would need to budget for silicone procurement.

The devices extend the literature on low-cost LMIC simulation^13^, ^15^ by addressing underrepresented procedural areas, providing fully replicable fabrication protocols (a frequent gap in published reports), and contributing evidence from an under-represented South Asian LMIC context.^16^

All devices were designed for replication by institutions with basic technical capability and local materials; no component requires specialist equipment beyond laser cutting and basic aluminium fabrication, both widely available commercially. Institutions are encouraged to substitute locally available alternatives, such as local synthetic fabric for felt layers, while retaining the core functional design.

A formal financial impact study was not possible within this reporting period, as not all simulators have completed one full financial year of use. Preliminary estimates suggest forgoing commercial equivalents saves approximately £13,595 in direct procurement costs, plus a further £700 from using these devices in participant-paid CPD workshops. In LMIC settings, this represents a meaningful redirection of resources rather than a marginal saving. Beyond direct savings, this low maintenance burden also frees resources otherwise spent on commercial servicing or licensing fees for additional teaching, trainee access, and patient care. The CIME Team plans formal educational validation studies, including pre/post confidence assessments, satisfaction surveys, and durability assessments in the future.

### Limitations

This is a technical innovation report and does not include quantitative educational outcome data; competency and retention studies are planned as separate future evaluations. Face and content validity were established through expert faculty review rather than formal validated instruments, and cost figures may vary by institution and country. No single commercial equivalent was identified for the C-Section Laparotomy Model or the Haemorrhoid Ligation Simulator, so their costs were calculated on a per-model basis using locally procured materials. Pilot testing of the BronchBox involved a small sample (n = 6); larger-scale validation is recommended before efficacy claims are made, and device durability under high-volume use has not yet been formally assessed.

## Conclusions

This project demonstrates that a dedicated team of technicians and clinicians can address unmet simulation training needs through innovative solutions that are safe, reusable, and contextually relevant. Despite their low cost, the trainers offered realistic tactile feedback and anatomical detail for hands-on practice. By making the design blueprints openly accessible, this work facilitates broader adoption and adaptation by other institutions, contributing to reducing inequities in simulation-based medical education. Beyond LMIC contexts, the fabrication principles and cost-reduction strategies described here may also support teaching innovation and sustainability goals within high-income country training programmes, including in the UK, where budget constraints and environmental considerations increasingly shape simulation resource planning.

## CRediT authorship contribution statement

**Muhammad Abbas Raza:** Writing – review & editing, Writing – original draft, Project administration, Methodology, Investigation, Formal analysis, Data curation, Conceptualization. **Mohammad Bilawal Khan:** Writing – review & editing, Writing – original draft, Visualization. **Ayaan Ali Qureshi:** Writing – review & editing, Writing – original draft, Visualization. **Ghalib Ali:** Software, Methodology, Investigation. **Ghansham Bhagwan Das:** Resources, Methodology, Investigation. **Abdul Ameen:** Writing – review & editing, Software, Methodology. **Selina Hasan:** Writing – review & editing, Supervision. **Faisal Wasim Ismail:** Writing – review & editing, Supervision, Resources, Conceptualization.

## Ethics approval and consent to participate

This study involved no human participants, patient data, biological specimens, or personal information. The study was reviewed by the Aga Khan University Ethics Review Committee and was granted exemption from ethical approval (ERC No. 2026-13130-42182).

## Informed consent

Not applicable. No human subjects were enroled and no personal or clinical data were collected; informed consent was therefore not required. In accordance with the journal's guidance that studies not requiring patient consent should state this explicitly, the authors confirm this is the case for the present project.

## Human subjects

This was a purely technical, device-development project. No human participants were enroled, recruited, or studied as research subjects, and no patient data, biological specimens, clinical records, or personal information were accessed or used at any stage of device design, fabrication, or reporting.

## Statement of compliance

All procedures associated with this project were performed in compliance with relevant institutional guidelines. As the project did not involve human participants, patient data, or biological specimens, it was reviewed by the Aga Khan University Ethics Review Committee (ERC) and granted exemption from full ethical review.

## Privacy of human subjects

The privacy rights of human subjects have been observed throughout. All photographs submitted for publication depict simulation devices and manikins only; no identifiable person appears in any image, and no patient consent for use of images was therefore required.

## Funding

This research did not receive any specific grant from funding agencies in the public, commercial, or not-for-profit sectors.

## Declaration of competing interest

The authors declare that they have no known competing financial interests or personal relationships that could have appeared to influence the work reported in this paper.

## Data Availability

The design specifications, fabrication protocols, and cost data for all simulation devices described in this study are included within the article. No additional datasets were generated or analysed during the current study. Further information and design blueprints are available from the corresponding author upon reasonable request.
